# Opposing function of mitochondrial prohibitin in aging

**DOI:** 10.18632/aging.100246

**Published:** 2010-12-16

**Authors:** Marta Artal-Sanz, Nektarios Tavernarakis

**Affiliations:** ^1^ Laboratory for Bioinformatics and Molecular Genetics, Bio III, Albert-Ludwigs-University of Freiburg, D-79104 Freiburg, Germany; ^2^Institute of Molecular Biology and Biotechnology, Foundation for Research and Technology, Heraklion, 71110, Crete, Greece

**Keywords:** insulin, lipids, metabolism, mitochondria, stress

## Abstract

While specific signalling cascades involved in aging, such as the insulin/IGF-1 pathway, are well-described, the actual metabolic changes they elicit to prolong lifespan remain obscure. Nevertheless, the tuning of cellular metabolism towards maximal survival is the molecular basis of longevity. The eukaryotic mitochondrial prohibitin complex is a macromolecular structure at the inner mitochondrial membrane, implicated in several important cellular processes such as mitochondrial biogenesis and function, molecular signalling, replicative senescence, and cell death. Recent studies in *C. elegans* have revealed that prohibitin differentially influences aging by moderating fat metabolism and energy production, in response to both intrinsic signalling events and extrinsic cues. These findings indicate that prohibitin is a context-dependent modulator of longevity. The tight evolutionary conservation and ubiquitous expression of prohibitin proteins suggest a similar role for the mitochondrial prohibitin complex during aging in other organisms.

## INTRODUCTION

Prohibitins are ubiquitous, evolutionarily strongly conserved proteins that localize to mitochondria. The eukaryotic mitochondrial prohibitin (PHB) complex comprises two highly homologous subunits, PHB-1 and PHB-2 (around 50% amino acid sequence identity and 60% similarity). PHB-1 and PHB-2, with molecular weights of 32 and 34 kDa respectively, associate to form a ring-like macromolecular structure of approximately 1 MD [1] with a diameter of 20-25 nm [2]. This high molecular weight complex has been identified inyeast, *Caenorhabditis elegans* and mammals [3-5]. PHB-1 and PHB-2 are interdependent for protein complex formation, and elimination of either PHB-1 or PHB-2 results in the absence of the whole PHB complex [3,6-8]. The PHB complex sits at the mitochondrial inner membrane facing the inter membrane space. However, detailed structural data about this highly conserved protein complex is lacking.

Several roles have been proposed for mitochondrial prohibitins. First, the PHB complex was suggested to regulate membrane protein degradation by the mitochondrial m-AAA protease [5]. Later, a function as a membrane-bound chaperone, which holds and stabilizes newly synthesized mitochondrial-encoded proteins was proposed [4,9]. PHB proteins might also play a role in stabilizing the mitochondrial genome [10-12]. In addition, the PHB complex has been implicated in mitochondrial morphogenesis by stabilizing OPA-1 [8], and by functioning as scaffold proteins that recruit membrane proteins to a specific lipid environment [13]. However, the true biochemical function of the mitochondrial prohibitin complex remains unknown. Accumulating evidence suggests that prohibitins function together within mitochondria [8,14-17] (reviewed in [9,18,19]), however, a number of diverse cellular functions have also been attributed to both PHB1 and PHB2 in other cellular compartments. These include a role in cell cycle progression, regulation of transcription and cell surface signaling (reviewed in [20-22]).

The high degree of evolutionary conservation of the PHB proteins (66/83% identity/similarity between human and *C. elegans*, and 53/79% identity/similarity between *C. elegans* and *Saccharomyces cerevisiae*) suggests an essential cellular function. Although disruption of the PHB complex in *S. cerevisiae* does not result in any observable growth phenotype under laboratory conditions [6,23], in *C. elegans* and in mice, prohibitins are required for embryonic development [3,7,8,24]. Post-embryonic depletion of prohibitins in *C. elegans* results in pronounced germline defects such as diminished oocyte production with smaller brood size [3], indicating that PHB proteins are specifically required in tissues that undergo cellular proliferation. Similarly, deletion of PHB2 in mouse embryonic fibroblasts results in severely impaired cellular proliferation [8]. Similarly, prohibitins are required for plant development [25,26] and are predominantly expressed in proliferating tissues [26]. PHB proteins are highly expressed in tissues that rely heavily on mitochondrial function and are particularly susceptible to mitochondrial dysfunction, including neurons, muscle, heart, liver, renal tubules, adrenal cortex, brown adipocytes and pancreatic islet cells [27]. Likewise, PHB proteins are expressed at high levels in mammalian proliferating cells, including neoplastic tissues [27,28], underscoring an essential role for prohibitins in regulating mitochondrial metabolism.

### Aging without prohibitin

Disruption of the PHB complex in *S. cerevisiae* decreases the replicative lifespan of yeast cells [6,23,29], which is accompanied by morphological changes characteristic of aging cells [23]. PHB depletion does not alter the chronological lifespan of non-dividing (G_0_-arrested) cells. However, *phb*-null mutants in stationary phase tend to lose respiratory capacity due to deletions of the mitochondrial genome (mtDNA) [30]. Loss of mtDNA can only be detected in old, non-dividing cells and not in young *phb*-null mother cells [6,31]. Similarly, defective mitochondrial segregation and aberrant mitochondrial morphology can only be detected in old *phb*-null mother cells at the end of their replicative lifespan [30]. This suggests that *phb*-null yeast cells undergo premature aging, probably due to a cumulative decline in cellular metabolic capacity.

In *C. elegans*, depletion of prohibitins by RNAi reduces the lifespan of wild type animals [17], recapitulating the yeast aging phenotype. PHB depleted animals develop slightly slower than wild type, are smaller in size and have severely reduced brood sizes. In addition, the reduced pharyngeal pumping rates and longer defecation cycles of PHB-deficient animals are indicative of metabolically compromised animals [3]. Surprisingly, however, PHB depletion markedly extends the lifespan of a large variety of *C. elegans* mutants [17]. These include reduced insulin/insulin growth gactor 1 (IGF-1) and transforming growth factor beta (TGF-β) signaling mutants, mutants with altered fat metabolism, mitochondrial electron transport chain (ETC) mutants and dietary restricted animals. It is interesting to note that these effects are the result of RNAi-mediated knockdown of prohibitin genes mainly in non-neuronal *C. elegans* cells, given that neurons are particularly refractory to RNAi[32]. Since neuroendocrine signaling is a key modulator of aging in diverse species, it would be particularly relevant to understand the role of neuronal prohibitins in the aging process. We discuss the implications of these findings below.

### Diapause signaling and prohibitin function

The Insulin/Insulin Growth Factor 1 (IGF-1) signaling pathway (IIS) regulates aging in worms, flies and mammals [33]. Mutations in the only *C. elegans* transmembrane insulin receptor kinase DAF-2 doubles the lifespan on wild type animals [34,35], and depletion of prohibitins further extends the lifespan of *daf-2* mutants by 150% [17]. The lifespan extension of *daf-2* mutants is dependent on the transcription factor DAF-16/FOXO [33]. Similarly, loss of DAF-16 fully suppresses the exceptional longevity of PHB-depleted *daf-2* mutants [17]. In addition to IGF signaling, the transforming growth factor beta (TGF-β) signal transduction pathway also controls aging in *C. elegans* [36]. Prohibitin depletion extends the lifespan of animals defective in TGF-β signaling, including the TGF-β homologue *daf-7* mutant and the transmembrane TGF-β receptor serine/threonine kinase *daf-4* mutant. The highly conserved IGF-1 and TGF-β signaling pathways also regulate diapause entry (dauer larva formation) in the nematode [35,37]. The transmembrane guanylate cyclase DAF-11, functions via both the insulin/IGF and the TGF-β pathways to modulate dauer formation [38]. Knock-down of *phb-1* or *phb-2* extends the lifespan of animals carrying a lesion in the *daf-11* gene. Thus, depending on the genetic background, prohibitin function has opposing effects on *C. elegans* aging. Although depletion of prohibitin compromises survival of wild-type animals, it substantially extends the lifespan of mutants defective in diapause signaling. Thus, the PHB complex plays an important role in modulating the rate of aging by coordinating mitochondrial energy metabolism in response to diapause signaling and energy demands.

Mitochondria are involved in key aging associated processes, such as cellular metabolism, ATP synthesis and the production and detoxification of reactive oxygen species (ROS). Unpaired mitochondrial function has been shown to increase lifespan across phyla [39]. However, the molecular basis of the effect of mitochondrial dysfunction in aging is poorly understood. PHB depletion by itself does not extend lifespan. However, it extends the lifespan of both, long-lived and short-lived, mitochondrial ETC defective animals. Knockdown of PHB genes extends the lifespan of *gas-1* mutants which carry a lesion in the 49-kDa iron-sulphur subunit of complex I of the ETC. Similarly, prohibitin depletion extends the lifespan of short-lived *mev-1* mutants, which are defective in succinate dehydrogenase cytochrome b, a component of complex II. Mutations in the *isp-1* gene, encoding the Rieske iron-sulfur subunit of complex III, and in the *clk-1* gene, involved in ubiquinone biosynthesis, extend the lifespan of *C. elegans* [29,40]. Prohibitin knockdown extends the lifespan of both, *isp-1* and *clk-1* mutant animals [17]. Therefore, reduced prohibitin activity promotes survival of animals with compromised mitochondrial function. Dietary restriction (DR) impacts on mitochondrial energy metabolism and on nutrient-sensing signaling pathways such as IIS and the target of rapamycin (TOR) signaling pathway [41]. Prohibitin deficiency improves the survival of dietary-restricted *eat-2* mutants. Whether PHB effects on longevity are mediated by the effects of DR on mitochondrial metabolism, on cellular signaling or on both is currently unknown.

Mitochondrial function is strongly linked to fat metabolism and altered fat metabolism accompanies the aging process. The *C. elegans* nuclear hormone receptor NHR-49 regulates fat mobilization by regulating the expression of a wide variety of genes, including mitochondrial fatty acid β-oxidation enzymes and fatty acid desaturases, such as FAT-7. Prohibitin deficiency extends the lifespan of both *nhr-49* and *fat-7* mutants [17]. Taken together, these findings indicate that prohibitin deficiency is beneficial for longevity in situations of altered growth factor signaling, defective mitochondrial function and altered fat metabolism.

All the above data indicates that the metabolic state will determine whether prohibitin will promote or compromise longevity. In agreement with this idea, a temperature shift is sufficient to revert the aging phenotype of PHB depletion. At higher temperature (25°C versus the standard 20°C growing conditions), where energy demands are higher and metabolic activity is elevated, prohibitin deficiency is beneficial for survival. Prohibitin deficiency is also beneficial under acute thermal stress (35°C), since elimination of PHB proteins render wild type animals strongly thermo-tolerant.

### Prohibitins and the regulation of metabolism

The link between mitochondrial energy metabolism and longevity has been most extensively studied in the nematode *C. elegans*. Many loss-of-function mutations in mitochondrial genes increase longevity [29,40,42-45]; in addition, genome-wide RNAi screens have identified many mitochondrial genes which reduction results in increased lifespan. These include mostly components of the ETC and ATP synthase, TCA cycle enzymes and mitochondrial carrier proteins [46-50]. Prohibitins, however, do not belong to the lifespan-extending class of mitochondrial genes, since disruption of PHB proteins shortens the lifespan of otherwise wild type animals. However, the remarkable increase in longevity that PHB depletion causes in metabolically compromised nematodes (e.g. IIS, mitochondrial ETC, fat metabolism and DR mutants) hints to an essential role of mitochondrial prohibitins in the regulation of cellular metabolism.

Prohibitin depletion results in reduced fat content, in all genetic backgrounds where PHB deficiency extends lifespan [17]. In wild type animals, depletion of PHB proteins also reduces fat content early during adulthood; however, this effect is lost in aged adults, where fat levels become indistinguishable from that of wild type animals. A notable difference exists in the case of IIS and TGF-β mutants where fat levels remain extraordinary low during aging upon PHB depletion. It is plausible that, in order to sustain reproduction, metabolically compromised PHB deficient animals generate energy by burning fat. However, impairment of fat accumulation cannot be excluded.

Mutations in the *nhr-49* gene result in fat accumulation due to the reduced expression of mitochondrial fatty acid β-oxidation enzymes and *nhr-49* mutants are short-lived. PHB knockdown in *nhr-49* mutants dramatically reduces fat content and extends longevity. NHR-49 regulates the expression of the delta-9 stearoyl-CoA desaturase FAT-7, which is required for the synthesis of monounsaturated fatty acids [51]. PHB depletion reduces fat content and extends the longevity of *fat-7* mutants. These observations suggest that prohibitin deficiency engages fat metabolism to promote longevity in sensitized genetic backgrounds.

How does PHB depletion affect mitochondrial activity to modulate aging? In wild type animals, prohibitin knockdown results in adult-onset mitochondrial over-proliferation in the intestine. This is plausibly due to the activation of a cellular retrograde response to mitochondrial deficiency, which has been shown in yeast to induce mitochondrial biogenesis [52]. However, in all cases where prohibitin depletion extends longevity, mitochondrial intestinal content is reduced compared to controls. Thus, there seems to be an inverse correlation between mitochondrial content and longevity in PHB depleted animals. One possible explanation is that mitochondrial biogenesis is switched on to compensate for the lack or prohibitins in wild type animals, which in turn leads to increased defective mitochondria, increased ROS production and shorter lifespan. However, the exact signals that inhibit mitochondrial biogenesis in all cases where PHB deficiency increases longevity are currently unknown.

Reduced fat and mitochondrial content upon PHB depletion correlate with extended longevity, however ATP content does not always correlate with extended longevity. Prohibitin knockdown significantly increases total ATP content in *daf-2*, *daf-7*, *isp-1* and *gas-1* mutants. However, no ATP increase is observed in *clk-1*, *eat-2* and *nhr-49* mutants. In particular, *eat-2* and *nhr-49* mutants depleted of prohibitins show less ATP content. Nevertheless, ATP content does not provide information about the capacity of ATP generation, since differences could be due to reduced or increased ATP consumption. ATP flux measurements should be much more informative.

### Oxidative stress and the requirement for prohibitins

To date, there is no clear mechanistic explanation for the observed increased longevity of mitochondrial mutants. Mitochondria are major producers of ROS as a result of electron misplacement along the ETC. The free radical theory of aging postulates that ROS cause aging by inflicting cellular damage. In view of this theory, one possibility is that mitochondrial mutations might result in reduced rate of living and decreased ROS production [29]. In other cases, ETC dysfunction might result in increased electron leakage and ROS production, which will consequently activate an adaptive hormetic response being ultimately beneficial for longevity [53]. Treatment with the free-radical scavenger N-acetyl-cysteine had no effect on longevity conferred by prohibitin knockdown in animals experiencing either oxidative stress (*mev-1* mutants) or thermal stress (wild-type nematodes grown at 25°C). Therefore, mitohormesis is unlikely to mediate the effects of prohibitin elimination on aging.

Although a handful of data support the oxidative damage theory of aging, recent data from diverse organisms put the correlation between oxidative stress and aging into question [54]. Prohibitin depletion results in some paradoxical outcomes regarding oxidative stress resistance. In agreement with the mitochondrial free radical theory of aging, wild type animals depleted of prohibitins live shorter, show increased ROS production and are more sensitive to exogenously added oxidative stress in the form of paraquat. Depletion of prohibitins in wild type animals results in increased mitochondrial content, which might translate into an increased number of sites where ROS are produce. The situation is more complex in the case of *daf-2(e1370)* mutants. Although old *daf-2(e1370)* adults are more resistant to paraquat upon PHB depletion, paraquat treatment results in the same level of ROS production in *daf-2(e1370)* adult animals regardless of the presence or the absence of the PHB complex. Moreover, surpriseingly, L4 *daf-2(e1370)* larvae are clearly more sensitive to paraquat in the absence of the PHB complex, despite the final output being extraordinarily increased lifespan.

Certainly, mitochondrial dysfunction upon prohibitin depletion results in more intricate physiological responses than increasing or reducing ROS levels and oxidative damage and unveiling the mechanisms implicated in mitochondrial-mediated life extension is crucial to understand how lifespan is regulated.

### Prohibitins and cellular signaling

A feasible mechanism involved in the lifespan extension conferred by prohibitin depletion is cellular signaling and the activation of alternative metabolic routes that will counter the mitochondrial defect. Long lived yeast mitochondrial mutants activate a retrograde signaling pathway that results in the activation of specific transcription factors that will shift metabolism away from the Krebs cycle towards the glyoxylate cycle. This metabolic shift has also been observed in dauer larvae and long-lived *daf-2* mutants [55,56]. It is possible that a metabolic shift also contributes to the extended lifespan of certain PHB-depleted *C. elegans* mutants. Although depletion of PHB does not affect the lifespan of the glyoxylate mutant *gei-7* (our unpublished observation) it would be interesting to see if *gei-7* is required in the mutant genetic backgrounds where PHB elimination extends lifespan.

It is generally believed that mitochondrial dysfunctions exert their effect on lifespan independently of the IIS pathway, because they show a synergistic effect with *daf-2* mutations and extend lifespan independently of DAF-16 [29,40,47,50]. However, some mitochondrial mutations require DAF-16 for lifespan extension and influence its nuclear localization [46,50]. Mitochondrial defects are associated with insulin resistance and diabetes and IIS regulates metabolism and the response to oxidative stress. Therefore, alteration of mitochondrial function is likely to affect longevity, through components of the IIS pathway. We have recently uncovered a surprising interaction of mitochondrial prohibitins with IIS in the regulation of longevity, which is dependent on the transcription factor DAF-16/ FOXO. However, the increased thermotolerance conferred by PHB-depletion is, at least partially, DAF-16/ FOXO independent, indicating that other pathways are involved in the metabolic changes elicited by prohibitin deficiency.

Reduced AMP/ATP ratios activate the AMP-activated protein kinase (AMPK). The *C. elegans aak-2*/AMPK is partially required for the life extension of *daf-2* and mitochondrial mutants [57,58]. The *aak-2*/AMPK kinase is required for the reduced fat content phenotype elicited by PHB depletion, and knockdown of prohibitins shortens the lifespan of *aak-2* mutants. On the other hand, *aak-2* mutants show increased mitochondrial biogenesis, which is further enhanced by PHB elimination [17]. AMPK targets p53 to promote cell survival under conditions of nutrient deprivation [59] and it has been suggested that cell cycle checkpoint control plays an important role in specifying longevity of mitochondrial mutants [60]. Prohibitin deficiency shortens the lifespan of p53 (*cep-1*) mutant animals, however, it would be necessary to examine the requirement of p53 in the cases where PHB deficiency extends lifespan.

The mitogen activated protein kinase (MAPK) JNK-1, is also required for the reduction of fat content in animals lacking prohibitins. On the one hand mitochondrial content is higher in *jnk-1* mutants and remains unchanged after prohibitin removal. On the other hand, overexpression of JNK-1 in wild-type animals reduces mitochondrial content and suppresses mitochondrial proliferation upon prohibitin depletion. *phb* gene knock-down shortens the lifespan of animals lacking JNK-1 and extends the lifespan of animals overexpressing JNK-1. Overexpression of JNK-1 promotes DAF-16/FOXO nuclear localization under conditions of stress [61]. Similarly, mutations in the Akt/PKB homologue AKT-1, which transduces insulin/IGF-1 signals [62], result in reduced mitochondrial content, which does not increase upon prohibitin depletion. AKT-1 mutations also promote DAF-16/FOXO nuclear localization, raising the possibility of DAF-16/FOXO being involved in the inhibition of mitochondrial biogenesis. Conversely, AKT-1 is not required for the reduction of fat content in *phb* deficient animals [17].

There is an urgent need to gain more insight into the transcriptional regulation and the signaling cascades involved in the regulation of lipid metabolism and mitochondrial biogenesis in response to PHB depletion in wild type animals and in the mutants where PHB depletion extends lifespan. This will allow delineating the molecular genetic pathways of PHB function as well as the molecular pathways involved in the crosstalk between mitochondria and cellular signaling networks in the regulation of aging.

## CONCLUSIONS

**Figure 1.**
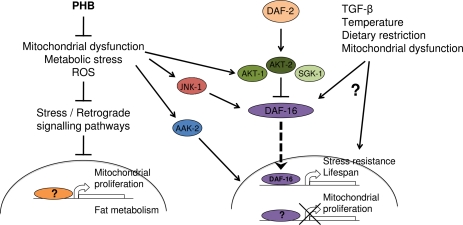
Opposing effects of prohibitin deficiency on aging Under normal conditions prohibitin promotes survival by moderating fat metabolism, mitochondrial proliferation and function, as well as, energy and ROS levels. In wild type animals, elimination of prohibitin results in mitochondrial defects and elicits a retrograde cellular response leading to mitochondria overproliferation and altered fat metabolism. In turn, accumulation of defective mitochondria lacking prohibitin results in increased reactive oxygen species production, metabolic defects, consequent cellular damage and reduced lifespan. Under reduced diapause signaling or under stress, where AKT/SGK-mediated inhibition of DAF-16/FOXO nuclear localization is relieved and stress kinases (AMPK, JNK) are activated, mitochondrial overproliferation is inhibited and cellular metabolism is adjusted towards fat utilization, promoting longevity.

Mitochondrial mutations result in pleiotrophic effects and possibly, different mutations will affect the aging rate differently and in a tissue specific manner. Aging is a hormonally regulated process; therefore, it is essential to understand when and in which specific tissues PHB depletion exerts its beneficial effects on longevity. The striking longevity phenotype observed upon prohibitin elimination in diapause mutants is, to our knowledge, the first example of a genetic manipulation with context-dependent, opposing effects on aging (Figure 1). Thus, prohibitins represent the first and only case, until now, of a protein that affects longevity in sharply opposing ways depending on the metabolic state. This indicates that important genes in the regulation of aging might have been overlooked because they only manifest their effects under certain conditions relevant to the aging process. Given the high degree of evolutionary conservation of prohibitins and the biochemical pathways involved in lifespan regulation, we anticipate a similar role for prohibitins in higher organisms.
